# Spruce Bark—A Source of Polyphenolic Compounds: Optimizing the Operating Conditions of Supercritical Carbon Dioxide Extraction

**DOI:** 10.3390/molecules24224049

**Published:** 2019-11-08

**Authors:** Petra Strižincová, Aleš Ház, Zuzana Burčová, Jozef Feranc, František Kreps, Igor Šurina, Michal Jablonský

**Affiliations:** 1 Institute of Natural and Synthetic Polymers, Department of Wood, Pulp and Paper, Faculty of Chemical and Food Technology, Slovak University of Technology in Bratislava, Radlinského 9, SK-812 37 Bratislava, Slovakia; ales.haz@stuba.sk (A.H.); igor.surina@stuba.sk (I.Š.); michal.jablonsky@stuba.sk (M.J.); 2 Institute of Food Science and Nutrition, Faculty of Chemical and Food Technology, Slovak University of Technology in Bratislava, Radlinského 9, SK-812 37 Bratislava, Slovakia; zuzana.burcova@stuba.sk (Z.B.); frantisek.kreps@stuba.sk (F.K.); 3 Institute of Natural and Synthetic Polymers, Department of Plastics and Rubber, Faculty of Chemical and Food Technology, Slovak University of Technology in Bratislava, Radlinského 9, SK-812 37 Bratislava, Slovakia; jozef.feranc@stuba.sk

**Keywords:** supercritical extraction, polyphenolic compounds, extractives, experimental design optimization, spruce bark

## Abstract

The present study described the optimization of the extraction process with carbon dioxide in supercritical state for obtaining extractives, especially polyphenols from softwood bark, Norway spruce (*Picea abies* (L.) Karst.). Using a full 2^3^ factorial design of experiments, the effect of varying the working parameters on the yield of extractives was studied for the following ranges: temperature 40–100 °C, pressure 1050–9000 psi (7.2–62 MPa), and concentration of EtOH/water co-solvent mixture 40–96.6%. In addition, total phenolics content and the antioxidant capacity of the spruce bark extract were determined. The optimum operating conditions for the yield of extractives were identified as 73 °C, 6465 psi (44.5 MPa), and 58% EtOH/water cosolvent concentration for a yield of 8.92%. The optimum conditions for achieving a total phenolics content of 13.89 mg gallic acid equivalent (GAE)/g dry extract were determined as: 45 °C, 1050 psi (7.2 MPa), and 96.6% EtOH/water mixture.

## 1. Introduction

Today, the increasing consumption of nonrenewable resources, such as oil, coal, and natural gas, is a worldwide problem. The supplies of these resources are limited and are expected to run out in a few years. The supply of natural gas is estimated to last for about 65 more years, while oil supply is expected to last 40 years and coal is expected to last 300 years. One of the solutions to avoid the exhaustion of fossil fuel is to find a sustainable method, using abundant resources, to obtain equivalent products—materials and chemicals made from lignocellulose materials. Currently, significant emphasis is placed on maximizing the exploitation of industrial biomass waste, such as tree bark, turning it into value-added products. Tree bark is gaining attention due to its unique composition and content of valuable biochemicals, i.e., extractives, and also as an economically and ecologically significant material due its biodegradability, renewability, low cost, and large potential availability [1]. Unfortunately, the wood processing industry does not currently use tree bark to its full potential. Most of the bark resulting from the wood, pulp, and paper industries is burnt for the production of energy and heat. The remaining part of the bark is used in traditional applications, e.g., as mulching material, in horticulture, and as ornamental bark [2,3].

The Slovak forest covers 41.1% of the country’s land area. The most abundant tree species in the Slovak republic include European beech (33.5%), Norway spruce (23.1%), sessile/English oak (10.6%), and Scots pine (6.8%) [1]. The industries log, on average, 5 Mm^3^ of Norway spruce per year, producing 0.5 Mm^3^ of spruce bark, considering a 10–12% volumetric bark content in logs. Since approximately 32% of the bark represents extractives, the valorization of this byproduct is highly important [4]. Such exploitation of the bark consists in extracting bark components, thus achieving higher value bio-based materials and bioactive compounds that could be used in a variety of pharmaceutical, chemical, cosmetic, and food applications. At the end of its lifecycle, bark could be combusted for generating electricity [5,6,7,8].

Extractives are derived from the metabolic processes of plants and include a wide range of phytochemicals with different physical properties. Spruce bark is rich in extractives, such as phenolic compounds, that make up one of the major families of secondary metabolites. In general, more than 8000 phenolic structures are currently known [9], including a diverse group of compounds of soluble phenolics such as flavonoids, phenolic acids, stilbenes, and nonsoluble compounds, such as lignins and condensed tannins [10]. Several studies have reported that these chemical compounds have a wide range of bioactive properties, such as cytotoxic, antibacterial, repellent, fungicidal, antimycotic, antitumor, anti-inflammatory, antiviral, antimalarial, antimutagenic, and growth-inhibiting effects [11,12,13,14,15,16,17]. The work of Jablonsky et al. [13] summarized the properties of 237 compounds extracted from coniferous bark and reported in various studies. The following activities have been established for the substances extracted from bark: Cytotoxic (25 identified substances), antioxidant (26 substances), fungicidal (20 substances), antibacterial (42 substances), anti-inflammatory (10 substances), antimutagenic (5 substances), pharmacokinetic (5 substances), pheromone (10 substances), and inhibiting (22 substances) [13]. For obtaining specific bioactive compounds with the desired properties, it is essential to find suitable experimental conditions of isolation, since the extraction parameters determine a qualitative and quantitative representation of individual components. The isolation method can provide a simple way to separate the desired extractives from components of the matrix while keeping the original matrix usable for ultimate energy production. Based on the literature survey, the following extraction methods have been investigated for phytochemicals: Solvent extraction ultrasound-assisted extraction (UAE) [18,19], supercritical fluid extraction (SFE) [20,21], microwave-assisted extraction (MAE) [22,23], accelerated solvent extraction (ASE) [24], and subcritical water extraction (SWE) [25,26].

The aim of this study was to optimize the working parameters (i.e., temperature, pressure, and cosolvent concentration) for isolating extractives from spruce bark using supercritical extraction with carbon dioxide (CO_2_).

The method of experimental design (DOE) was applied for statistical evaluation of the measurements. These measurements were used for optimizing the extraction conditions to achieve the maximum extraction yield from spruce bark. In addition, the isolated extractives were determined to evaluate their total phenolics content and their antioxidant capacity. Based on the findings of this study, we concluded that the selected extraction conditions (pressure, temperature, and concentration of the solvent), as well as their interaction with other parameters, have a significant impact on the monitored properties.

## 2. Results and Discussion

In this study, the method of experimental design was applied to investigate the effects of temperature, pressure and cosolvent concentration on the yield of extractives. In addition, total phenolics content and the antioxidant capacity was assessed. A full 2^3^ factorial design of experiments was performed for a comprehensive description of the selected physical parameters affecting the extraction process. The effects of the selected variables were determined in the following ranges: temperature t = 40–100 °C, cosolvent concentration c = 40–96.6% and pressure p = 1050–9000 psi (7.2–62 MPa). The extraction time (50 min), particle size (1–1.4 mm) and the ratio between the raw material and cosolvent (1:5 (*w*/*v*)) were kept constant during the experimental procedures. The complete DOE consisted of twenty combinations, including six replicates of the center point for the reported standard deviation from the overall mean. The levels of the independent variables under investigation are listed in Table 1 and Table 2.

The method of experimental design mathematically evaluates of measurement using analysis of variance. The analysis of variance (ANOVA) was employed to assess the statistically significant factors. The mathematical relationship of each response to the selected variables is approximated by the general quadratic polynomial shown in Equation (1):

Y = β_0_ + ∑β_j_X_j_ + ∑β_jj_X_j_^2^ + ∑β_jk_X_j_X_k_ (1)

where Y is the response, β_0_ is a constant, β_j_ is the linear coefficient, β_jk_ is the interaction or crossed coefficient, and β_jj_ is the quadratic coefficient [27].

The output of the method of experimental design is a system of regression equations that are used for optimization of the system. Statistical and analysis data were evaluated using the STATIS software [27] and for optimisation of yield of extractives was used subprogram Solver, MS Excel.

First of all, the effect of temperature was examined between 40–100 °C. The minimum extraction temperature (T_min_ = 40 °C) was defined in the vicinity of the critical point of carbon dioxide (T_c_ CO_2_ = 31.3 °C) and the maximum temperature was set at T_max_ = 100 °C to avoid possible degradation of thermally labile compounds, which occurs during heating [28,29]. Ethanol was used as co−solvent in various concentrations to extract polar compounds, whereas pure carbon dioxide is useful in the extraction of non-polar to slightly polar compounds. Ethanol is recognized safe, with low environmental impact, and due it is low toxicity it is currently used in food and nutraceutical extraction processes [30,31,32]. According Monrad et al. [33], and Santos et al. [34], a lower concentration of ethanol cosolvent allows obtaining a higher yield of extractives. Still, a wide concentration range of the ethanol cosolvent was examined for assessing its effect on the yield of the extractives. The maximum value of ethanol was limited to its affordable concentration (96.6%). Regarding the maximum and minimum values of pressure (1050–9000 psi), they were established according to the limits of the equipment. The particle size of the matrix also has an important impact on the overall extraction efficiency. The particle size of spruce bark in the range 1–1.4 mm, as used for the experiment, was determined based on our previous research [23,35]. The obtained results have shown that, to achieve a higher yield, particle size should be greater than 0.3 mm and smaller than 2 mm. In this respect, it has been established that the surface area increases as the particle size decreases, due to better solute-solvent interaction [36]. A particle size greater than 2 mm offers a smaller surface area for mass transfer, compared with a particle size smaller than 2 mm. On the other hand, a particle size smaller than 0.3 mm leads to lower yield of extractives because of particle can agglomerated in an extractor [37].

### 2.1. Yield of Extractives

The general equation describing the yield of extractives as a function of the variables is shown in Equation (2).

YE = b_0_ + b_1_x_1_ + b_2_x_2_ + b_3_x_3_ + b_12_x_1_x_2_ + b_13_x_1_x_3_ + b_23_x_2_x_3_ + b_11_x_12_ + b_22_x_22_ + b_33_x_32_ (2)

Table 3 and Table 4 present the results obtained by ANOVA analysis.

**Figure 1:**
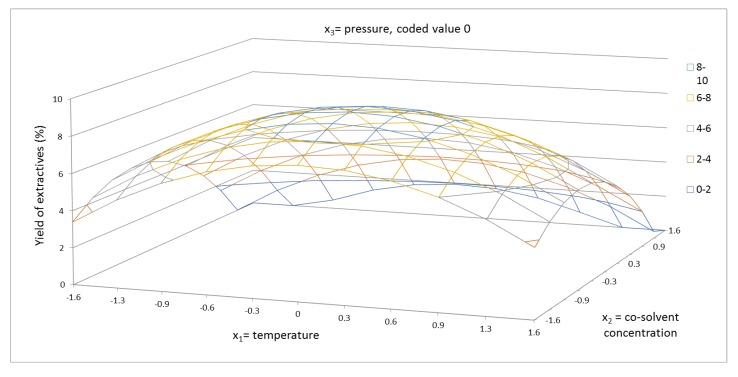
Response surface of extractives yield (%) as a function of temperature in range of 40–100 °C and cosolvent concentration of 40–96.6% EtOH/water, in coded values, at constant pressure of 5025 psi (coded value 0).

According to the results obtained, both linear parameters b_1_ and b_2_ and both interaction parameters b_11_ and b_22_ strongly influenced the yield of extractives. Figure 1 shows the response surface of the yield of extractives for temperature and cosolvent concentration. The response surface passed through the global maximum. In this case, it is interesting that the lowest cosolvent concentration led to a higher value of yield of extractives compared to YE, which was achieved by the highest cosolvent concentration. These findings are in agreement with those reported in previous investigations [33,34]. This behavior proves the significant role of the cosolvent polarity, also confirmed by our regression analysis results, which indicated that the linear terms of cosolvent concentration played the main role in the system. In Figure 1, the strong interaction for both factor at medium temperature and at medium cosolvent concentration is shown. This effect is explained by the dissolution of valuable compounds in the system. An increase in the temperature caused a decrease in the surface tension and viscosity of the system, increasing the hydrophilicity of target analytes, hence the dissolving power was higher [38]. Thus, temperature was evaluated to be the second most important factor influencing the yield of extractives, as can be noted in Table 4. Higher temperature stimulated the diffusion of carbon dioxide and the fluidity of the membrane increased. It improved the isolation of cellular material from the cells [39,40,41]. However, overly high temperature can lead to sample agglomeration. The yield of extractives at the highest temperature (100 °C, 68.3%, 5025 psi) used in this study was 4.54%, which is twice lower compared to that reached at the temperature of 70 °C (68.3%, 5025 psi, YE = 8.7%). The results listed in Figure 1 also point out that the amount of ethanol was a significant parameter. In fact, its effect was more important than that of increasing the temperature by several degrees Celsius. Ethanol enhanced the yield of extractives more than twofold.

**Figure 2:**
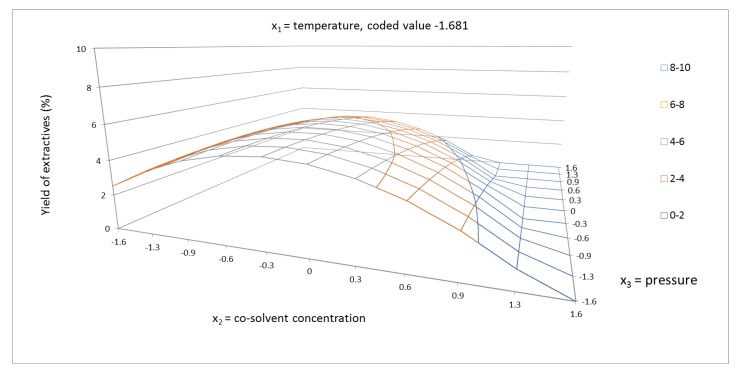
Response surface of extractives yield (%) as a function of cosolvent concentration of 40–96.6% EtOH/water and pressure in the range of 1050–9000 psi, in coded values, at constant temperature of 70 °C (coded value −1.681).

**Figure 3:**
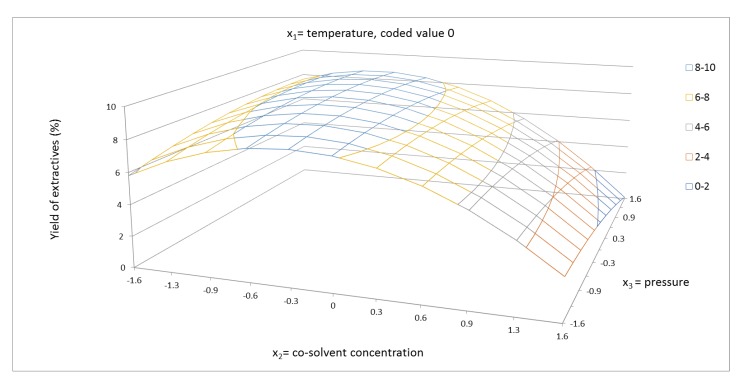
Response surface of extractives yield (%) as a function of cosolvent concentration of 40–96.6% EtOH/water and pressure in the range of 1050–9000 psi, in coded values, at constant temperature of 70 °C (coded value 0).

**Figure 4:**
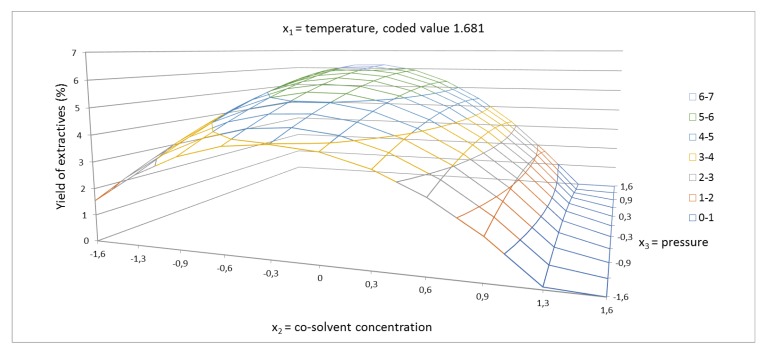
Response surface of extractives yield (%) as a function of cosolvent concentration of 40–96.6% EtOH/water and pressure in the range of 1050–9000 psi, in coded values, at constant temperature of 70 °C (coded value + 1.681).

**Figure 5:**
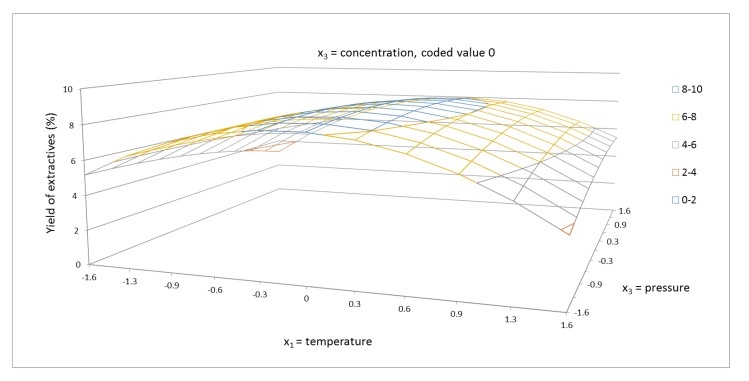
Response surface of extractives yield (%) as a function of temperature, in the range of 40–100 °C and pressure in the range of 1050–9000 psi, in coded values, at constant EtOH/water cosolvent concentration of 68.3% (coded value 0).

The response surface for the yield of extractives of cosolvent concentration and pressure is shown in coded values from −1.681 to +1.681 (Figure 2, Figure 3 and Figure 4). In these figures, the synergic effect between cosolvent concentration and pressure is shown. As can be seen, at temperature 100 °C, the yield of extraction increased with increasing pressure, but at temperature 40 °C, the yield of extractives decreased with increasing pressure. The pressure as a single factor has been evaluated as factor with minor positive effect on the yield of extractives. In addition to the linear term of pressure, the quadratic term b_22_ had a minor effect on the system. The synergic effect between temperature and pressure, as shown in Figure 5, was also confirmed.

### 2.2. Total Phenolics Content and Antioxidant Capacity of Extracts

**Figure 6:**
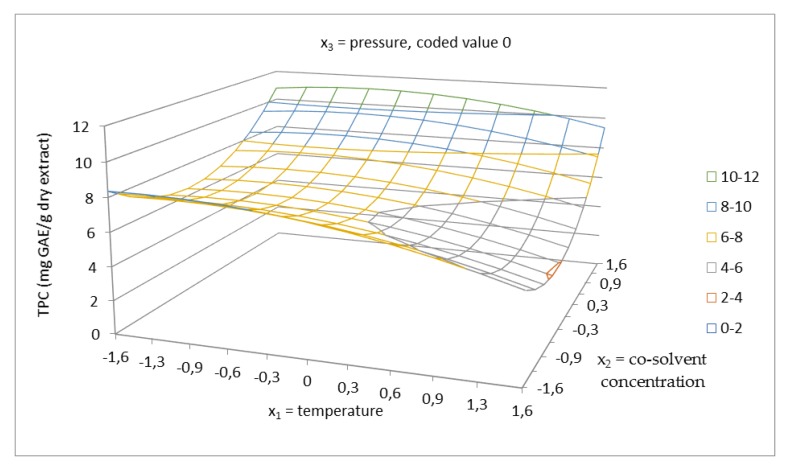
Response surface of total phenolics content as a function of temperature in the range of 40–100 °C and EtOH/water cosolvent concentration of 40–96.6% in coded values at constant pressure of 5025 psi (coded value 0).

**Figure 7:**
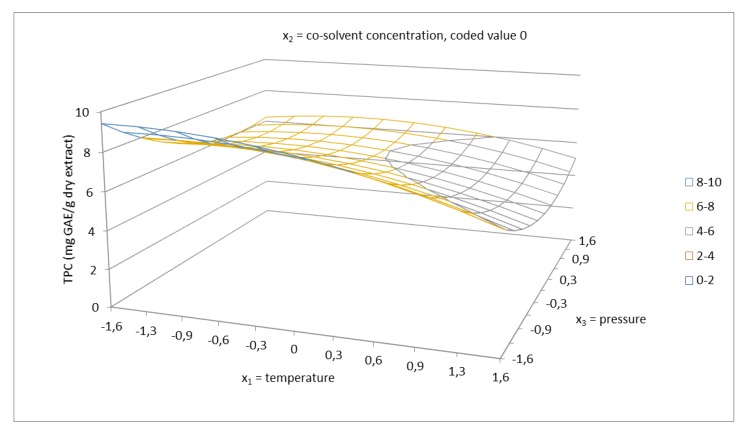
Response surface of total phenolics content as a function of temperature in the range of 40–100 °C and pressure in the range of 1050–9000 psi in coded values at constant EtOH/water cosolvent concentration of 68.3% (coded value 0).

**Figure 8:**
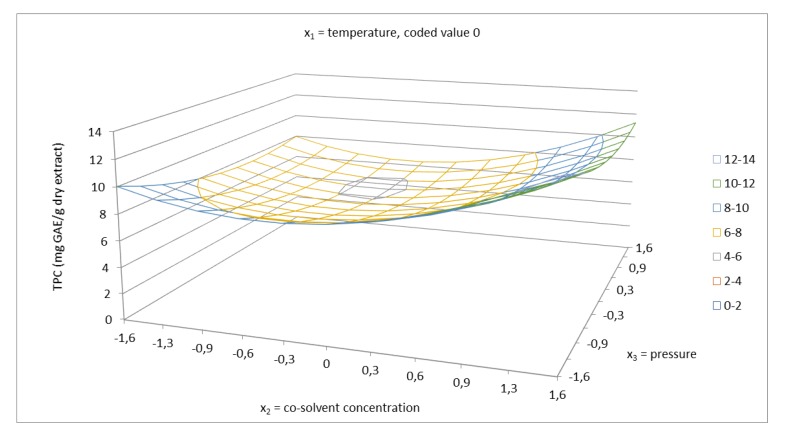
Response surface of total phenolics content as a function of EtOH/water cosolvent concentration of 40–96.6% and pressure in the range of 1050–9000 psi in coded values at constant temperature of 70 °C (coded value 0).

Phenolic compounds represent a large group of molecules with a variety of functions in plants. Some are used as antioxidants to prevent the oxidation of fatty acids. The ability of an antioxidant to scavenge DPPH radicals is attributed to their hydrogen donating ability [42,43]. Total phenolics content was evaluated using a calibration curve obtained with gallic acid and expressed in mg GAE/g dry extract. The regression equation from the standard curve was y = 0.142x + 0.0142 (R^2^ = 0.9994). The antioxidant capacity of the extracts was quantified using a calibration curve obtained with Trolox. The regression equation from standard curve was y = −0.0099x + 0.8607 (R^2^ = 0.9947). The obtained data for TPC and the antioxidant capacity were determined for each sample and the results are presented in Table 2. As can be seen, the total phenolics content of the extracts was in the range of 4.41–11.03 mg GAE/g dry extract and the antioxidant capacity was in the range of 0.68–0.79 µM TE/mg dry extract. The visualization of the effect of working parameters on total phenolics content, using 3D response surface plots, is presented in Figure 6, Figure 7 and Figure 8. In this case, based on the ANOVA results in Table 4, the linear terms of all the independent variables had a positive effect on TPC. From the highest to the lowest, the influencing parameters were ranked in the following order: Cosolvent concentration, temperature, and pressure. According to the results, the interaction of factors, namely, cosolvent concentration–pressure, also exerted a strong influence on total phenolics content. The other interaction of factors had a minor effect on TPC. Cosolvent concentration was confirmed to have the main effect on the system, with temperature having the other significant effect. The response surface of TPC as a function of temperature and cosolvent concentration at a constant pressure 5025 psi is shown in Figure 6. According to the results obtained, using lower temperature gives higher total phenolics content. At the temperature 100 °C, total phenolics content of 4.47 mg GAE/g dry extract was reached, which is about 2.9 mg lower than that achieved at the temperature of 40 °C (7.38 mg GAE/g dry extract). These results can be attributed to the thermal degradation of polyphenols using higher temperature. According to the literature [44], rutin degrades sharply above to 100 °C. Figure 8 shows the interaction between cosolvent concentration and pressure. Both factors showed a strong interaction at high co-solvent concentration and at high pressure, as well as at high cosolvent concentration and low pressure. In addition, the relationship between TPC and TEAC was discussed. From the obtained result, the correlation between TPC and TEAC was not found, since the temperature has a significant effect on the stability of polyphenols and their biological activities. According to the literature [44], the degradation of flavonoids depends on structural solidity and the modifications of structure of polyphenols lead to changes in antioxidant capacity.

### 2.3. Extraction Optimization

The operating conditions for supercritical extraction with carbon dioxide were determined in order to obtain the maximum yield of extractives. The optimal conditions for extractives yield in the ranges of the selected parameters were found as follows: 73 °C, 58% EtOH/water, 6465 psi (44.5 MPa), for which the statistically validated regression models provided a yield of extractives of 8.92%. The standard error was ± 0.2736 %. In addition, in the study, the optimum conditions for TPC were determined. According to the statistical calculations, the optimum conditions for TPC (13.89 mg GAE/g dry extract) were limited to value 45 °C, 96.6%, and 1050 psi, whereas the maximum value of TPC was not reached. The standard error for TPC was ± 0.3014 %.

## 3. Materials and Methods

### 3.1. Chemicals

All the reagents, standards, and solvents were of analytical grade and were purchased from Sigma-Aldrich (Saint-Louis, MO, USA), Centralchem (Bratislava, Slovak Republic), VWR International (Radnor, PA, USA), and Alfa Aesar (Haverhill, MA, USA). Carbon dioxide of 99.5% purity was supplied by Messer Tatragas (Bratislava, Slovak Republic).

### 3.2. Plant Materials

Spruce bark (*Picea abies*) was provided as an industrial waste by the timber company Bioenergo Ltd. (Ruzomberok, Slovakia). The spruce bark was air-dried until a constant weight was reached, homogenized by grinding using a knife mill with a motor power of 7.5 kW, and separated into different fractions using sieves. The spruce bark fraction of 1–1.4 mm was extracted using a supercritical extraction with carbon dioxide and analyzed to determine the content of holocellulose (52.0 ± 0.2%), lignin (26.4 ± 1.3%), ash (3.6 ± 0.4%), and extractives (12.7 ± 0.01%). The moisture content of the material (8.77 ± 0.08%) was determined by drying approximately 1 g of spruce bark at 105 °C for 6 h until complete moisture removal according to ISO 3130:1975 [45].

### 3.3. Supercritical Extraction with CO_2_

**Figure 9:**
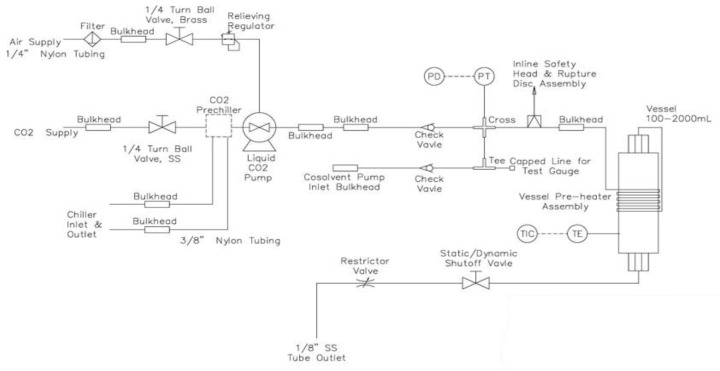
Diagram of supercritical extraction equipment [49].

The extraction was performed using a Supercritical Fluid Extractor model SFT-150 from Supercritical Fluid Technologies, Inc., Newark, NJ, USA. The schematic diagram of the SFT-150 is shown in Figure 9. Supercritical extraction has several advantages over conventional extraction techniques, e.g., the elimination of polluting organic solvents and reduced product contamination, thus being an environment-friendly, safe, fast, and cost-effective technique to yield high-purity products [36,38]. Carbon dioxide was used as supercritical fluid. As a supercritical fluid, carbon dioxide manifests higher diffusion coefficients and lower viscosities than a liquid solvent. Consequently, the diffusivity and solubility in such fluids tend to be much higher than in compared with liquids, resulting in fast reaction kinetics. Besides carbon dioxide is generally recovered as a safe, nontoxic, nonflammable, low-cost and high-purity solvent, which can easily reach supercritical conditions [46,47,48].

Approximately 40 g of air-dried samples were filled into the extractor vessel, together with the cosolvent (mixture of ethanol and water, V = 200 mL). Then, the extraction conditions were set according to the Design of Experiments. The samples were extracted in the static mode with pure CO_2_ for 50 min.

### 3.4. Determination of Extractives Yield

The yield of extractives (YE, %) in each experiment was determined by drying the bark samples at 105 °C to a constant weight. The results are expressed on the basis of the dry matter weighed before and after extraction, as shown in Equation (3):

YE (%) = 100 × (m_i_ − m_j_)/m_i_ (3)

where m_i_ is the dry mass (g) of the bark before extraction and m_j_ is the mass (g) of the bark after extraction and drying.

### 3.5. Determination of Total Phenolics Content

Total phenolics content (TPC) of the extracts was determined by the Folin-Ciocalteu assay, based on the redox reactions of phenolic compounds [50]. A volume of 0.5 mL of Folin-Ciocalteu reagent and 0.5 mL of the extract or ethanol (as blank sample) were pipetted into a test tube. After 3 min, 1.5 mL of 20% sodium carbonate solution and distilled water were added into the test tube. After stirring, the mixture was incubated in a closed dark colored flask at room temperature for 120 min, and then the absorbance of the solution was recorded at 765 nm. The TPC in the extracts was determined using the calibration curve based on the absorbance at 765 nm and expressed as gallic acid equivalent (GAE) in mg per 1 g of dry extract.

### 3.6. Determination of Antioxidant Capacity

**Figure 10:**
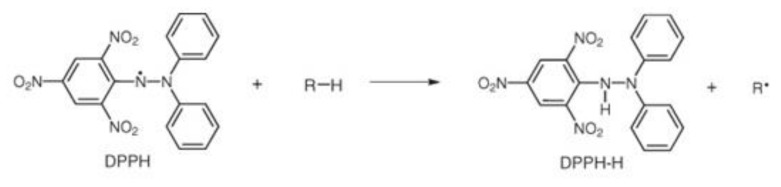
Reaction mechanism of 2,2-diphenyl-1-picrylhydrazyl (DPPH) radical with an antioxidant [52].

The antioxidant activity of the extracts was determined using the Trolox equivalent antioxidant capacity (TEAC) method using 2,2-diphenyl-1-picrylhydrazyl (DPPH; C_18_H_12_N_5_O_6_, M = 394.33 g/mol) as a free radical Figure 10. This assay is based on the ability of the antioxidant to neutralize the DPPH radical via electron donation. The odd electron of the nitrogen atom in the DPPH radical is reduced by receiving a hydrogen atom from the antioxidant to the corresponding hydrazine. The DPPH radical produces deep purple-colored solutions. The purple color of the initial solution turns yellow when the free radical is blocked by the substance that can donate a hydrogen atom [51].

This assay was performed according to the method modified by Mareček at al. (2017) [53]. An amount of 200 μL sample (1 mg of dry extract diluted in 10 mL of 95% ethanol), mixed with 75 μL of DPPH (0.5 mM in 95% ethanol) or 75 μL of ethanol (as blank sample), was used for the measurement at 517 nm. It was determined by the calibration curve that the concentration of Trolox ranged from 0.5–80 μM. The antioxidant activity was expressed as Trolox equivalents (μM TE) on 1 mg of dry extract.

## 4. Conclusions

Based on the analysis of the experiment, our findings lead to the conclusion that the extraction conditions have a significant impact on the extraction yield, especially the cosolvent concentration and temperature used. The cosolvent concentration had the most significant effect on the yield of extractives, followed by the temperature, while the pressure had a minimal effect on the yield of extractives. Higher temperature enabled an easier passage of the target analytes through the pores on the surface of the natural matrix. The use of ethanol and water as SFE modifier is suitable to extract a significant share of valuable compounds from spruce bark, containing of polyphenols, which were found to exhibit antioxidant capacity. Results also reveal that the antioxidant capacity of the spruce bark extract cannot be defined solely its total phenolics content. Therefore, the antioxidant properties depend on the sample and differ among the phenolic compounds. Moreover, the amount of all the phenolics occurring in spruce bark still remains unknown, requiring further detailed studies.

## Figures and Tables

**Table 1:** The experimental design conditions.

Name of Factors	Factors	Coded Variable Level				
		−1.682	−1	0	1	1.682
**Temperature (°C)**	x_1_	40	52	70	88	100
**Cosolvent concentration (%)**	x_2_	40.0	51.5	68.3	85.1	96.6
**Pressure (psi)**	x_3_	1050	2660	5025	7390	9000

**Table 2:** Coded and real values of design of experiment (DOE).

Run	Coded Levels of Factors			Real Levels of Factors					
	x_1_	x_2_	x_3_	t (°C)	c (%)	p (psi)	YE (%)	TPC ^a^	TEAC ^b^
**1**	−1	−1	−1	52.16	51.47	2661.74	7.78	6.61	0.75
2	1	−1	−1	87.84	51.47	2661.74	6.68	4.41	0.75
3	−1	1	−1	52.16	85.13	2661.74	4.14	9.22	0.71
4	1	1	−1	87.84	85.13	2661.74	4.94	8.46	0.74
5	−1	−1	1	52.16	51.47	7388.26	7.80	5.82	0.74
6	1	−1	1	87.84	51.47	7388.26	9.20	5.24	0.79
7	−1	1	1	52.16	85.13	7388.26	3.73	9.23	0.75
8	1	1	1	87.84	85.13	7388.26	3.99	8.46	0.69
9	−α	0	0	40.00	68.30	5025.00	3.78	7.39	0.76
10	α	0	0	100.00	68.30	5025.00	4.54	4.47	0.75
11	0	−α	0	70.00	40.00	5025.00	5.70	10.32	0.76
12	0	α	0	70.00	96.60	5025.00	2.15	9.61	0.70
13	0	0	α	70.00	68.30	1050.00	7.82	11.30	0.74
14	0	0	α	70.00	68.30	9000.00	6.99	5.46	0.75
15	0	0	0	70.00	68.30	5025.00	8.14	6.24	0.74
16	0	0	0	70.00	68.30	5025.00	8.69	6.40	0.74
17	0	0	0	70.00	68.30	5025.00	8.71	6.50	0.75
18	0	0	0	70.00	68.30	5025.00	8.70	6.10	0.74
19	0	0	0	70.00	68.30	5025.00	8.19	6.34	0.73
20	0	0	0	70.00	68.30	5025.00	8.67	6.83	0.74

−α = −1.682, α = 1.682, ^a^ TPC in mg GAE/g dry extract; ^b^ TEAC in µM TE/ mg dry extract.

**Table 3:** Analysis of variance for yield of extractives.

Source of Variability	s	f	s ^ 2	F	F_c_
s_1_	29.89	3	9.96	133.01	5.41
s_2_	51.84	6	8.64	115.34	4.95
S_E_	0.37	5	0.07	1.00	-
S_LF_	6.76	5	1.35	18.05	5.05

**Table 4:** Regression analysis for yield of extractives and total phenolics content.

Coefficients	Yield of Extractives (%)		TPC (mg GAE/g Dry Extract)	
	b_i_	b_c_	b_i_	b_c_
b_0_	8.49	0.29	6.30	0.32
b_1_	0.25	0.19	−0.67	0.21
b_2_	−1.46	0.19	0.89	0.21
b_3_	−0.07	0.19	−0.68	0.21
b_11_	−1.33	0.19	−0.33	0.20
b_12_	0.00	0.25	0.16	0.27
b_13_	0.33	0.25	0.20	0.27
b_22_	−1.42	0.19	1.10	0.20
b_23_	−0.4	0.25	−0.004	0.27
b_33_	−0.19	0.19	0.49	0.20

The statistically significant regression coefficients are pointed in bold b_c—_The critical value of the coefficient on 95% probabilistic level.

## References

[1] Kemppainen K., Siika-aho M., Pattathil S., Giovando S. (2014). Spruce bark as an industrial source of condensed tannins and non-cellulosic sugars. Ind. Crops Prod..

[2] Harkin J.M., Rowe J.W. United States Forest Service research note Forest Products Laboratory. https://www.fpl.fs.fed.us/documnts/fplrn/fplrn091.pdf.

[3] Miranda I., Gominho J., Mirra I., Pereira H. (2012). Chemical characterization of barks from Picea abies and Pinus sylvestris after fractioning into different particle sizes. Ind. Crops Prod..

[4] Räisänen T., Athanassiadis D. Semanticscholar. https://www.semanticscholar.org/paper/Basic-chemical-composition-of-the-biomass-of-pine%2C-R%C3%A4is%C3%A4nen-Athanassiadis/f17423a8129685dd0a874ad47d16431862505d7b.

[5] Pietarinen S.P., Willför S.M., Ahotupa M.O., Hemming J.E., Holmbom B.R. (2006). Knotwood and bark extracts: Strong antioxidants from waste materials. J. Wood Sci..

[6] Conde E., Cadahia E., Diez-Barra R., García-Vallejo M. (1996). Polyphenolic composition of bark extracts fromEucalyptus camaldulensis, *E. globulus* and *E. rudis*. Holz als Roh-und Werkstoff.

[7] Şen A., Miranda I., Santos S., Graça J., Pereira H. (2010). The chemical composition of cork and phloem in the rhytidome of *Quercus cerris* bark. Ind. Crops Prod..

[8] Valentín L., Kluczek-Turpeinen B., Willför S., Hemming J., Hatakka A., Steffen K., Tuomela M. (2010). Scots pine (*Pinus sylvestris*) bark composition and degradation by fungi: Potential substrate for bioremediation. Bioresour. Technol..

[9] Tsao R.J.N. (2010). Chemistry and biochemistry of dietary polyphenols. Nutrients.

[10] Ozcan T., Akpinar-Bayizit A., Yilmaz-Ersan L., Delikanli (2014). Phenolics in human health. Int. J. Chem. Eng. Appl..

[11] Nascimento M., Santana A., Maranhão C., Oliveira L., Bieber L., Chamy R., Rosenkranz F. (2013). Phenolic extractives and natural resistance of wood. Biodegradation-Life of Science.

[12] Laurova M., Vybohova E., Mamonova M. (2007). Heartwood and Sapwood Liphophilic Extractives of Oak (*Quercus petraea* (Mattusch.) Liebl.). Acta Facultatis-Xylologiae.

[13] Jablonsky M., Nosalova J., Sladkova A., Haz A., Kreps F., Valka J., Miertus S., Frecer V., Ondrejovic M., Sima J.J.B.a. (2017). Valorisation of softwood bark through extraction of utilizable chemicals. A review. Biotechnol. Adv..

[14] Caron A., Altaner C.M., Gardiner B., Jarvis M.C. (2013). Distribution of extractives in Sitka spruce (*Picea sitchensis*) grown in the northern UK. Eur. J. Wood Wood Prod..

[15] Hon D.N.-S., Shiraishi N. (2000). Wood and cellulosic chemistry, revised, and expanded.

[16] Kanadaswami C., Lee L., Lee P., Hwang J., Ke F., Huang Y., Lee M. (2005). The antitumor activities of flavonoids. In Vivo.

[17] Middleton E., Kandaswami C., Theoharides T.C. (2000). The effects of plant flavonoids on mammalian cells: Implications for inflammation, heart disease, and cancer. Pharmacol. Rev..

[18] Ghitescu R.-E., Volf I., Carausu C., Bühlmann A.-M., Gilca I.A., Popa V.I. (2015). Optimization of ultrasound-assisted extraction of polyphenols from spruce wood bark. Ultrason. Sonochem..

[19] Lazar L., Talmaciu A.I., Volf I., Popa V.I. (2016). Kinetic modeling of the ultrasound-assisted extraction of polyphenols from *Picea abies* bark. Ultrason. Sonochem.

[20] Talmaciu A.I., Volf I., Popa V.I. (2015). Supercritical Fluids and Ultrasound Assisted Extractions Applied to Spruce Bark Conversion. Environ. Eng. Manage. J..

[21] Co M., Fagerlund A., Engman L., Sunnerheim K., Sjöberg P.J., Turner C. (2012). Extraction of antioxidants from spruce (*Picea abies*) bark using eco-friendly solvents. Phytochem. Anal..

[22] Bouras M., Chadni M., Barba F.J., Grimi N., Bals O., Vorobiev E. (2015). Products. Optimization of microwave-assisted extraction of polyphenols from Quercus bark. Ind. Crops Prod..

[23] Sládková A., Benedeková M., Stopka J., Šurina I., Ház A., Strižincová P., Čižová K., Škulcová A., Burčová Z., Kreps F. (2016). Yield of polyphenolic substances extracted from spruce (*Picea abies*) bark by microwave-assisted extraction. BioResources.

[24] Jablonsky M., Vernarecová M., Ház A., Dubinyová L., Skulcova A., Sladková A., Surina I.J. (2015). Extraction of phenolic and lipophilic compounds from spruce (*Picea abies*) bark using accelerated solvent extraction by ethanol. Wood Res..

[25] Talmaciu A.I., Ravber M., Volf I., Knez Ž., Popa V.I. (2016). Isolation of bioactive compounds from spruce bark waste using sub-and supercritical fluids. J. Supercrit. Fluids.

[26] Belwal T., Ezzat S.M., Rastrelli L., Bhatt I.D., Daglia M., Baldi A., Devkota H.P., Orhan I.E., Patra J.K., Das G. (2018). A critical analysis of extraction techniques used for botanicals: Trends, priorities, industrial uses and optimization strategies. TrAC Trends Anal. Chem..

[27] Alexy P., Viselka M. (1998). STATIS Program for Planning and Evaluation of Experiments Food Technology.

[28] Destandau E., Michel T., Elfakir C. (2013). Microwave-assisted extraction. Natural Product Extraction: Principles and applications.

[29] Xiao W., Han L., Shi B. (2008). Microwave-assisted extraction of flavonoids from Radix Astragali. Sep. Purif. Technol..

[30] Markom M., Hasan M., Daud W.R.W., Singh H., Jahim J., Technology P. (2007). Extraction of hydrolysable tannins from *Phyllanthus niruri* Linn.: Effects of solvents and extraction methods. Sep. Purif. Technol..

[31] Katayama S., Zhao L., Yonezawa S., Iwai Y. (2012). Modification of the surface of cotton with supercritical carbon dioxide and water to support nanoparticles. J. Supercrit. Fluids.

[32] Leitão N., Prado G., Veggi P., Meireles M., Pereira C. (2013). *Anacardium occidentale* L. leaves extraction via SFE: Global yields, extraction kinetics, mathematical modeling and economic evaluation. J. Supercrit. Fluids.

[33] Monrad J.K., Howard L.R., King J.W., Srinivas K., Mauromoustakos A. (2010). Subcritical solvent extraction of anthocyanins from dried red grape pomace. J. Agric. food chem..

[34] Santos S.A., Villaverde J.J., Silva C.M., Neto C.P., Silvestre A.J. (2012). Supercritical fluid extraction of phenolic compounds from *Eucalyptus globulus* Labill bark. J. Supercrit. Fluids.

[35] Strizincova P., Ház A., Sládková A., Jablonský M., Surina I. Optimization conditions of extraction. Proceedings of the FP1306 COST Action Third Workshop and Fourth MC Meeting.

[36] Mandal S.C., Mandal V., Das A.K. (2015). Essentials of botanical extraction: Principles and applications.

[37] Baldosano H.Y., Castillo M.B.M.G., Elloran C.D.H., Bacani F.T. Effect of particle size, solvent and extraction time on tannin extract from Spondias purpurea bark through soxhlet extraction. Proceedings of the DLSU Research Congress, De La Salle University.

[38] Sapkale G., Patil S., Surwase U., Bhatbhage P. (2010). Supercritical fluid extraction. Int. J. Chem. Sci..

[39] Garcia-Gonzalez L., Geeraerd A.H., Spilimbergo S., Elst K., Van Ginneken L., Debevere J., Van Impe J., Devlieghere F. (2007). High pressure carbon dioxide inactivation of microorganisms in foods: The past, the present and the future. Int. J. Food Microbiol..

[40] Wimmer Z., Zarevúcka M. (2010). A review on the effects of supercritical carbon dioxide on enzyme activity. Int. J. Mol. Sci..

[41] Čolnik M., Primožič M., Knez Ž., Leitgeb M. (2016). Use of non-conventional cell Disruption Method for extraction of Proteins from Black Yeasts. Front. Bioeng. Biotechnol..

[42] Liu J., Jia L., Kan J., Jin C.-H. (2013). In vitro and in vivo antioxidant activity of ethanolic extract of white button mushroom (*Agaricus bisporus*). Food chem. Toxicol..

[43] Kraujalis P., Venskutonis P.R., Ibáñez E., Herrero M. (2015). Optimization of rutin isolation from *Amaranthus paniculatus* leaves by high pressure extraction and fractionation techniques. J. Supercrit. Fluids.

[44] Chaaban H., Ioannou I., Chebil L., Slimane M., Gérardin C., Paris C., Charbonnel C., Chekir L., Ghoul M. (2017). Effect of heat processing on thermal stability and antioxidant activity of six flavonoids. J. Food process. Preserv..

[45] International Standard ISO. https://www.iso.org/standard/8288.html.

[46] Zougagh M., Valcárcel M., Rıos A. (2004). Supercritical fluid extraction: A critical review of its analytical usefulness. TrAC Trends Anal. Chem..

[47] Veggi P.C., Prado J.M., Bataglion G.A., Eberlin M.N., Meireles M.A.A. (2014). Obtaining phenolic compounds from jatoba (*Hymenaea courbaril* L.) bark by supercritical fluid extraction. J. Supercrit. Fluids.

[48] Martinez-Correa H.A., Magalhães P.M., Queiroga C.L., Peixoto C.A., Oliveira A.L., Cabral F.A. (2011). Extracts from pitanga (*Eugenia uniflora* L.) leaves: Influence of extraction process on antioxidant properties and yield of phenolic compounds. J. Supercrit. Fluids.

[49] Supercritical Fluids. http://www.supercriticalfluids.com/wp-content/uploads/Spec-Sheet-SFT-150.pdf.

[50] Yu L., Haley S., Perret J., Harris M. (2002). Antioxidant properties of hard winter wheat extracts. Food chem..

[51] Kedare S.B., Singh R. (2011). Genesis and development of DPPH method of antioxidant assay. J. Food Sci. Technol..

[52] Gressler V., Moura S., Flores A.F., Flores D.C., Colepicolo P., Pinto E. (2010). Antioxidant and antimicrobial properties of 2-(4, 5-dihydro-1H-pyrazol-1-yl)-pyrimidine and 1-carboxamidino-1H-pyrazole derivatives. J. Braz. Chem. Soc..

[53] Mareček V., Mikyška A., Hampel D., Čejka P., Neuwirthová J., Malachová A., Cerkal R. (2017). ABTS and DPPH methods as a tool for studying antioxidant capacity of spring barley and malt. J. Cereal Sci..

